# Evidence for the Complexity of MicroRNA-Mediated Regulation in Ovarian Cancer: A Systems Approach

**DOI:** 10.1371/journal.pone.0022508

**Published:** 2011-07-21

**Authors:** Shubin W. Shahab, Lilya V. Matyunina, Roman Mezencev, L. DeEtte Walker, Nathan J. Bowen, Benedict B. Benigno, John F. McDonald

**Affiliations:** 1 School of Biology, Georgia Institute of Technology, Atlanta, Georgia, United States of America; 2 Parker H. Petit Institute for Bioengineering and Bioscience, Georgia Institute of Technology, Atlanta, Georgia, United States of America; 3 Ovarian Cancer Institute, Atlanta, Georgia, United States of America; 4 Department of Biological Sciences, Clark Atlanta University, Atlanta, Georgia, United States of America; Roswell Park Cancer Institute, United States of America

## Abstract

MicroRNAs (miRNAs) are short (∼22 nucleotides) regulatory RNAs that can modulate gene expression and are aberrantly expressed in many diseases including cancer. Previous studies have shown that miRNAs inhibit the translation and facilitate the degradation of their targeted messenger RNAs (mRNAs) making them attractive candidates for use in cancer therapy. However, the potential clinical utility of miRNAs in cancer therapy rests heavily upon our ability to understand and accurately predict the consequences of fluctuations in levels of miRNAs within the context of complex tumor cells. To evaluate the predictive power of current models, levels of miRNAs and their targeted mRNAs were measured in laser captured micro-dissected (LCM) ovarian cancer epithelial cells (CEPI) and compared with levels present in ovarian surface epithelial cells (OSE). We found that the predicted inverse correlation between changes in levels of miRNAs and levels of their mRNA targets held for only ∼11% of predicted target mRNAs. We demonstrate that this low inverse correlation between changes in levels of miRNAs and their target mRNAs *in vivo* is not merely an artifact of inaccurate miRNA target predictions but the likely consequence of indirect cellular processes that modulate the regulatory effects of miRNAs *in vivo.* Our findings underscore the complexities of miRNA-mediated regulation *in vivo* and the necessity of understanding the basis of these complexities in cancer cells before the therapeutic potential of miRNAs can be fully realized.

## Introduction

MicroRNAs (miRNAs) are members of an abundant class of small (∼22 nts) regulatory RNAs believed to play significant roles in a variety of biological processes and diseases in both plants and animals [1]. Of recent interest, is the possible contribution of aberrantly expressed miRNAs to cancer initiation and development [2], [3]. Numerous *in vitro* studies have demonstrated that miRNAs are capable of inhibiting the translation and/or facilitating the degradation [4], [5], [6], [7] of their targeted mRNAs making them attractive candidates for potential use in cancer therapy [8], [9]. However, the potential clinical utility of miRNAs in cancer therapy rests heavily upon our ability to understand and accurately predict the consequences of fluctuations in levels of miRNAs within the context of tumor cells *in vivo*. The general expectation that changes in levels of miRNAs will be inversely correlated (IC) with changes in levels of their mRNA targets [10], [11], [12], [13], [14] has yet to be conclusively tested within the context of tumor cells *in vivo.* For example, previous independent estimates of relative miRNA levels in ovarian cancers vs. controls often have been inconsistent, possibly due to differences in sample type (*e.g*., bulk tissue samples vs. micro-dissected cells, etc.), biological variability among different cancer sub-types and individual patient samples (*e.g*., [15], [16], [17], [18], [19], [20]) or due to inaccuracies in the prediction of the mRNA targets [21], [22].

In an effort to reduce variation that may obscure biologically significant trends, we have conducted microarray (Affymetrix) analyses of miRNAs and mRNAs from the same ovarian cancer epithelial (CEPI) cells isolated from patient samples by laser capture micro-dissection (LCM). We monitored differences in levels of miRNA expression between CEPI and ovarian surface epithelial (OSE) cells (collected from ovaries of normal patients) with expression levels of their putative mRNA targets as determined by various prediction algorithms and by experimental validation. While ovarian cancers may arise from either the fimbrial epithelium of the oviduct or OSE, it has recently been shown that both classes of cells are part of a transitional epithelium of common origin and thus either may serve as a precursor to CEPI [23]. Since OSE can be harvested from the surface of ovaries with minimal contamination, they were selected as appropriate controls in our study.

We found that only ∼11% of mRNA targets displaying significant (p<0.005) changes in levels of expression in CEPI relative to normal were IC with changes in levels of their regulating miRNAs (p<0.01). The levels of the majority (∼79%) of target mRNAs were unchanged in CEPI while the rest (∼10%) of the mRNA targets displayed changes in levels positively correlated (PC) with their respective regulating miRNAs. We conclude that the low predictability of miRNA regulatory effects in CEPI isolated from patient samples is attributable to the complexity of miRNA function *in vivo*.

## Results

### The majority of miRNAs differentially expressed in CEPI relative to OSE are up-regulated

**Figure 1:**
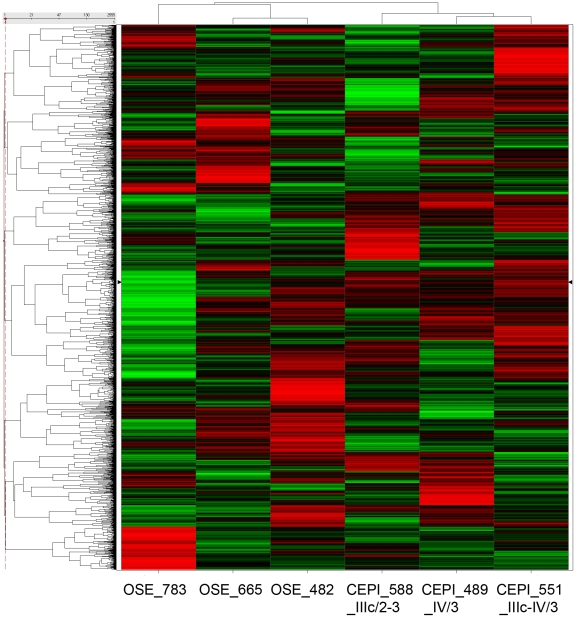
Unsupervised hierarchical clustering of CEPI and OSE samples based on probesets expressed on the Ambion miRChip. An unsupervised hierarchical clustering of the CEPI and OSE samples was carried out using all detected probesets on the Ambion miRChip array, regardless of differential expression. Probesets with standard deviation <0.5 across all samples were removed prior to clustering. The clustering shows that the CEPI and OSE samples cluster into separate groups, which suggests the variance between the groups is greater than that within the groups.

**Figure 2:**
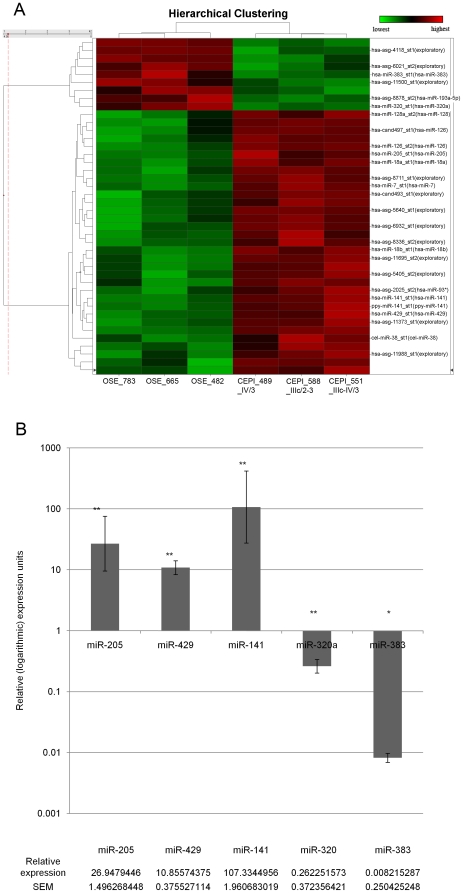
Differentially expressed miRNAs in CEPI cells from ovarian cancer patients relative to OSE. (A) Hierarchical clustering of normal and cancer patient samples based on differentially expressed miRNAs between CEPI cells and OSE cells (p<0.01, ≥2fold change). The dendogram on the left shows that the probes cluster into two groups corresponding to the up-regulated and down-regulated miRNAs differentially expressed between normal and cancer. IDs of selected probes are given on the right (including the annotated human miRNAs, see Table S1 for a complete list). Key: hsa-miR-x: annotated human miRNAs; hsa-asg/cand-x: predicted candidate human miRNAs; ppy-: *Pongo pygmaeus* miRNA; cel-: *C. elegans* miRNA. (B) Confirmation of expression patterns for selected miRNAs by qPCR. The relative expression of each miRNA in logarithmic units in cells from 3 cancer patients is shown compared to cells from 3 normal ovaries after normalization to RNU6B (ΔΔCt method). These were found to be statistically significant by Relative Expression Software Tool (REST®) by a randomization method. Randomization was performed 5000 times. Consistent with the microarray results, miR-141, miR-205 and miR-429 were confirmed to be significantly up-regulated in cancer, while miR-320 and miR-383 were found to be significantly down-regulated. Error bars represent standard error of the mean.

Unsupervised hierarchical clustering of the expression profiles of miRNAs detected on the miRChip (Asuragen Inc, Austin, TX) was performed on three CEPI and three OSE patient samples (Figure 1; see Table 1 for clinical information regarding samples). The miRChip contains a total of 13,349 probes including 467 annotated human miRNAs (Sanger miRBase V9.2 [24], [25], [26]), 455 miRNAs annotated in various other species and 12,894 (exploratory) probes of predicted, but as yet not validated/annotated miRNAs. Using a threshold of 2-fold or greater change, 42 miRNA probes were found to be differentially expressed (p<0.01) between our cancer and control samples. Of these, 33 were up-regulated and 9 down-regulated in the CEPI relative to OSE, including 12 previously annotated human miRNAs (9 up-regulated and 3 down-regulated). A heat map of the 42 differentially expressed miRNA probes is presented in Figure 2a (the miRNA sequences of these 42 probes, log_2_ difference between CEPI and OSE, and p-values from t-test are provided in Table S1). To independently test the validity of the differential miRNA expression patterns determined by microarray, we conducted measurements of miRNA levels using quantitative (real-time) polymerase chain reaction (qPCR). Five (*miR-141, miR-429, miR-205, miR-383 and miR-320*) of the 12 previously annotated human miRNAs shown to be differentially expressed by microarray were selected for qPCR analysis in three cancer and three control samples. The qPCR results confirmed the differences detected in the microarray study (Figure 2b).

**Table 1:** Clinical information of patient samples used in this study.

Patient ID	Ovarian Histopathology	Stage/Grade	Age at Surgery (years)
551a,b,c	papillary serous carcinoma	IIIc/IV/3	59
588a,b,c	papillary serous carcinoma	IIIc/2-3	71
489a,b	papillary serous carcinoma	IV/3	48
620^c^	papillary serous carcinoma	III/IV/3	62
434^a^	within normal limits	N/A	41
440^a^	within normal limits	N/A	50
475^a^	within normal limits	N/A	63
470^a^	Ov-within normal limits; hx of endometrial ca.	Ov-N/AEndo-1b/1	44
437^a^	Ov-within normal limits; hx of cervical ca.	Ov-N/ACerv-1b/3	54
482^b^	within normal limits	N/A	44
665^b^	within normal limits	N/A	84
783b,c	within normal limits	N/A	52
838^c^	within normal limits	N/A	51
846^c^	within normal limits	N/A	51

a mRNA microarray;

b microRNA microarray;

c real-time PCR.

Clinical information relevant to this study for each patient is shown. In addition, a legend is given to identify which patient was used for each microarray and qPCR experiment. Abbreviations used- Cerv: Cervix; Endo: Endometrium; Ov: Ovary; ca.: Carcinoma; hx: History.

In contrast to some earlier studies [15], [16], [18], [19], [20], our results indicate that the majority of miRNAs displaying significant differences in levels of expression between CEPI and OSE are up-regulated in ovarian cancer. The basis of this discrepancy is unknown but may be due to differences in sample type (e.g., bulk tissue samples vs. micro-dissected cells, etc.) and/or biological variability among individual patient samples. Our finding that the majority of miRNAs are up-regulated in ovarian cancer is consistent with the fact that miRNA targets are significantly enriched (hypergeometric distribution, p<0.05) among mRNAs down-regulated in our cancer samples, while up-regulated genes have relatively few miRNA targets (Figure S1; heat maps showing hierarchical clustering of the samples using all probesets is presented in Figure S2 and using only differentially expressed mRNAs in Figure S3; a list of all differentially expressed mRNAs is presented in Table S2). A possible biological explanation of why miRNAs are up-regulated in CEPI may lie in the fact that in contrast to other cancers [27], [28], the transition from OSE to CEPI is postulated to involve changes from a less differentiated to a more differentiated state [29], [30], [31].

Among the 12 previously annotated miRNAs displaying a significant change in levels in the CEPI samples, miR-205, miR-141, and miR-429 are all significantly up-regulated consistent with previous studies linking these miRNAs with the maintenance of cells in the differentiated epithelial state [32]. Of the miRNAs that are down-regulated in CEPI relative to OSE, miR-320 and miR-383 are located in regions associated with frequent DNA copy number losses in ovarian cancer [19]. miR-320 previously has been identified as an inhibitor of lung carcinoma proliferation [33]. Its down-regulation in CEPI suggests that it may act as a tumor suppressor in ovarian cancers as well.

### Only ∼11% of the predicted mRNA targets of miRNAs differentially expressed between the CEPI and OSE display the expected inverse pattern of change in gene expression

Previous studies have established that human miRNAs repress gene expression by pairing with complementary sequences located within the 3′ untranslated regions (3′ UTR) of targeted mRNAs resulting in translational repression and mRNA degradation [6], [7]. Based on these findings, changes in the expression levels of miRNAs are generally predicted to be inversely correlated with changes in the expression levels of their targeted mRNAs [10], [11], [12], [13], [14]. To test this hypothesis in the context of cells isolated from patient samples, we compared changes in the levels of the previously annotated human miRNAs with levels of their predicted target mRNAs in our CEPI samples relative to the OSE controls. The putative mRNA targets of these previously annotated human miRNAs were initially identified using the miRanda algorithm [34], [35], [36]. The results indicate that only ∼11% of the changes in mRNA expression between CEPI and OSE were inversely correlated (IC) with the observed changes in miRNA levels (Tables 2 & S3). Earlier studies of levels of miRNAs and their targeted mRNAs in a series of cell lines reported similar findings [13], [37], [38]. The expression of the majority (No Change, NC: ∼79%) of the predicted mRNA targets was not significantly different (p>0.005 and/or fold change <2) between the CEPI and OSE samples, while ∼10% of the targeted mRNAs displayed changes positively correlated (PC) with their putatively regulating miRNAs.

**Table 2:** Summary values of IC, PC and NC targets in CEPI vs. OSE using miRanda.

miRNA	Total Targets (miRanda)	Inversely Correlated Targets (%)	No Change (%)	Positively Correlated Targets (%)
miR-7	2363	10.62	79.94	9.44
miR-18a	1738	11.10	79.86	9.03
miR-18b	1710	11.40	79.24	9.36
miR-126	84	14.29	75.00	10.71
miR-128	2691	11.82	79.23	8.96
miR-141	3074	13.53	77.91	8.56
miR-205	2268	13.49	78.31	8.20
miR-429	3316	13.48	78.62	7.90
miR-93*	2252.00	13.14	77.00	9.86
Average (for up-regulated miRNAs)	2166.22	12.54	78.34	9.11
miR-383	2118	9.02	78.80	12.18
miR-320a	3073	8.30	79.08	12.63
miR-193a-5p	1216	12.01	78.45	9.54
Average (for down-regulated miRNAs)	2135.67	9.77	78.78	11.45
Average (for Up and Down)	2150.94	11.16	78.56	10.28

miRNA targets that were differentially expressed between CEPI and OSE based on t-test p<0.005 and fold change of at least 2 were classified as being IC or PC with their regulating miRNAs (while target genes that do not meet the above criteria are classified as NC). Total number of targets from miRanda algorithm present after removing probe sets with “Absent” calls in all samples is shown along with fraction of IC, PC and NC targets for each miRNA. On average, 78.6% of the target mRNAs are “No Change”, or do not change significantly with miRNAs, 11.2% are “inversely correlated” and 10.3% are “positively correlated” with miRNAs.

To determine if the unexpectedly low percentage of IC changes may be a computational artifact of our use of the miRanda algorithm to predict targeted mRNAs, we repeated the analysis independently using the PicTar (www.pictar.org) and TargetScan (www.targetscan.org) miRNA target prediction algorithms. In both instances, the results were consistent with our original finding that only a minority of the changes in mRNA expression between CEPI and OSE are inversely correlated (IC) with the observed changes in miRNA levels (PicTar: IC 9.4%, PC 10.1%, NC 80.5%; TargetScan: IC 10.4%, PC 10.3%, NC 79.3%; see Tables 3, S4 & S5). To increase the stringency of target predictions, we reanalyzed the data using only miRNA targets that were commonly predicted by all three algorithms (intersection). We found that by overlapping the three independent prediction algorithms, each miRNA had fewer predicted targets, yet using these commonly predicted targets, only ∼7% of the mRNA changes between CEPI and OSE were found to be inversely correlated (IC) with the observed changes in miRNA levels (Table 4). We also analyzed our data using intersections of predictions from two algorithms at a time (miRanda+TargetScan, miRanda+PicTar, and PicTar+TargetScan), and again we found that changes in levels of only 6–9% of the predicted mRNA targets were inversely correlated with changes in miRNA levels (Tables S6, S7 and S8).

**Table 3:** Summary of average IC, PC and NC fractions based on miRanda, PicTar and TargetScan predicted targets.

	Inversely Correlated Targets (%)	No Change (%)	Positively Correlated Targets (%)
miRanda	11.16	78.56	10.28
TargetScan (TS)	10.37	79.34	10.29
PicTar (PT)	9.41	80.48	10.11
Average of different algorithms	10.31	79.46	10.23

Average fraction of IC, NC and PC targets in CEPI vs. OSE calculated for all 12 annotated miRNAs using miRanda, TargetScan (TS) or PicTar (PT) (See Tables 2, S4 and S5 for details). The average IC, NC and PC fractions of the mean values calculated from these 3 different algorithms are also shown.

**Table 4:** Summary values of IC, PC and NC targets in CEPI vs. OSE using overlap of miRanda, TargetScan and PicTar target predictions.

miRNA	Total Targets (M_TS_PT)	Inversely Correlated Targets (%)	No Change (%)	Positively Correlated Targets (%)
miR-7	105	10.48	84.76	4.76
miR-18a	59	13.56	83.05	3.39
miR-18b	59	13.56	83.05	3.39
miR-126	2	0.00	100.00	0.00
miR-128	237	11.81	79.75	8.44
miR-141	177	15.82	74.58	9.60
miR-205	73	21.92	67.12	10.96
Average (for up-regulated miRNAs)	101.71	12.45	81.76	5.79
miR-383	15	0.00	80.00	20.00
miR-320a	112	3.57	84.82	11.61
miR-193a-5p	2	0.00	50.00	50.00
Average (for down-regulated miRNAs)	43.00	1.19	71.61	27.20
Average (for Up and Down)	72.36	6.82	76.68	16.50

miRNA targets that were differentially expressed between CEPI and OSE based on t-test p<0.005 and fold change of at least 2 were classified as being IC or PC with their regulating miRNAs (while target genes that do not meet the above criteria are classified as NC). Total number of targets from the intersection (M_TS_PT) of miRanda (M), TargetScan (TS) and PicTar (PT) predictions present after removing probe sets with “Absent” calls in all samples is shown along with fraction of IC, PC and NC targets for each miRNA. On average, 76.7% of the target mRNAs are “No Change”, or do not change significantly with miRNAs, 6.8% are “inversely correlated” and 16.5% are “positively correlated” with miRNAs.

Experimental validation is currently considered the most stringent method to validate miRNA targets [39], [40]. Thus, to further test the possibility that the low inverse correlation between changes in miRNA levels and target mRNAs observed in the tissue samples was merely a reflection of the limited accuracy of target prediction algorithms, we conducted a series of transfection experiments using two miRNAs that were significantly up-regulated in CEPI (miR-7 and miR-128) to identify experimentally validated targets of these miRNAs in CEPI.

A series of independent transfection experiments was carried out with miR-7, miR-128 and a matched set of negative control miRNAs in a well-established ovarian cancer cell line (HEY) [41]. To determine the effectiveness of our transfections (positive controls), we monitored expression levels of two previously confirmed mRNA targets of miR-7 (epidermal growth factor receptor, EGFR [42] and miR-128 (B lymphoma Mo-MLV insertion region 1 homolog, BMI1 [43]) regulation. The results confirm the effectiveness of both transfections (Figure S4). RNA was collected from cells after transfection and the relative levels of mRNAs present were determined by Affymetrix microarray analyses (HG-U133 Plus 2.0; Tables S9 and S10).

Consistent with the results from CEPI tissue samples, only a minority of predicted mRNA targets were found to be significantly reduced (IC) after miR-7 or miR-128 transfection (Table 5). The predicted mRNA targets that were significantly down-regulated in these transfection experiments were taken as “experimentally validated” mRNA targets of miR-7 and miR-128 respectively. Using only these targets, we reanalyzed the microarray data from the tissue samples and found that only ∼8% (range 0–18%) of the “experimentally validated” mRNA targets displayed changes in expression IC with changes in miR-7 and miR-128 expression in CEPI (Table 6). Collectively our results indicate that the low inverse correlation between changes in levels in miRNAs and their target mRNAs *in vivo* is not merely an artifact of inaccurate target predictions but rather a reflection of the complexity of miRNA function in cancer cells.

**Table 5:** Summary values of IC, PC and NC targets in transfection experiments using miRanda, TargetScan and PicTar target predictions.

miRNA_prediction algorithm	Total Targets	Inversely Correlated Targets (%)	No Change (%)	Positively Correlated Targets (%)
	miR-7 transfection			
miR-7_M	2432	7.98	91.24	0.78
miR-7_TS	259	23.55	75.29	1.16
miR-7_PT	238	16.39	83.19	0.42
miR-7_M_TS	234	20.51	78.63	0.85
miR-7_PT_TS	118	22.88	77.12	0
miR-7_M_PT	159	20.75	79.24	0
miR-7_M_TS_PT	99	23.23	76.77	0
	miR-128 transfection			
miR-128_M	2744	9.55	79.15	11.30
miR-128_TS	654	16.36	73.85	9.79
miR-128_PT	441	15.19	74.60	10.20
miR-128_M_TS	449	20.04	72.83	7.13
miR-128_TS_PT	301	16.94	73.75	9.30
miR-128_M_PT	306	17.32	74.18	8.50
miR-128_TS_M_PT	221	19.91	73.30	6.79

miRNA targets that were differentially expressed between miR-7 or miR-128 transfected cells compared to negative control miRNA transfected cells, based on fold change of at least 1.4 and false discovery rate threshold of 5% were classified as being IC or PC with their regulating miRNAs (while target genes that do not meet the above criteria are classified as NC). Total number of targets from miRanda (M), TargetScan (TS) and PicTar (PT) or their intersections (two at time and all three) present after removing probe sets with “Absent” calls in all samples is shown along with fraction of IC, PC and NC targets for each miRNA.

**Table 6:** Summary values of IC, PC and NC mRNAs in CEPI vs. OSE for miR-7 and miR-128 using “experimentally validated” targets only.

miRNA_prediction algorithm	Total Targets	Inversely Correlated Targets (%)	No Change (%)	Positively Correlated Targets (%)
miR-7_M	180	12.22	80.56	7.22
miR-7_TS	60	6.67	88.33	5
miR-7_PT	37	0	91.89	8.11
miR-7_M_TS	47	6.38	87.23	6.38
miR-7_PT_TS	27	0	96.30	3.70
miR-7_PT_M	33	0	90.91	9.09
miR-7_M_TS_PT	23	0	95.65	4.35
Average for miR-7 (all methods)	58.14	3.61	90.12	6.26
miR-128_M	252	11.90	74.60	13.49
miR-128_TS	103	10.68	75.73	13.59
miR128_PT	63	17.46	69.84	12.70
miR-128_M_TS	87	12.64	72.41	14.94
miR-128_PT_TS	49	16.33	69.39	14.29
miR-128_M_PT	52	11.54	75	13.46
miR-128_M_PT_TS	43	11.63	72.09	16.28
Average for miR-128 (all methods)	92.71	13.17	72.73	14.11
Average (overall)	75.43	8.39	81.42	10.19

Targets that were predicted by miRanda (M), TargetScan (TS), PicTar (PT) or any combination of the 3 programs and were down-regulated (FDR<5%, fold change≥−|1.4|) in miR-7 transfection or in miR-128 transfection were assumed to be experimentally validated targets. The direction of change of these same targets in CEPI vs. OSE were then used to calculate IC, NC and PC fractions using a p-value<0.005 and a fold change of at least 2 in the tissue microarray data.

## Discussion

It is well known that genes and their mRNA products are subject to a vast array of regulatory controls. The relative contribution of malfunctions in these controls to the onset and progression of diseases, such as cancer, can be varied and complex [44]. miRNAs and other small non-coding RNAs recently have been shown to be important regulators of gene expression, and disruption of miRNA expression has been implicated in many diseases including cancer [3], [45], [46], [47], [48]. While it is well established that miRNAs can serve as useful biomarkers for the diagnosis and staging of a variety of human cancers (e.g., [2], [49], [50]), the manner and extent to which miRNAs contribute to the processes underlying cancer is only beginning to be understood [2], [45]. For example, the regulatory function of miRNAs was initially believed to operate exclusively at the translational level [51], [52], but more recent findings have demonstrated that these small regulating RNAs also play a role in the modulation of mRNA stability and that these two modes of control may be inter-related [7], [53]. We still do not rule out the possibility that regulation of some target genes may occur at the translational level without significant changes in mRNA level, and therefore may have been ignored in our analyses.

Extensive *in vitro* and *in vivo* studies previously have demonstrated that human miRNAs can repress gene expression by pairing with sequences located within the 3′ untranslated regions of targeted messenger RNAs (mRNAs) resulting in translational repression and subsequent mRNA degradation [1], [6], [7]. Thus far, there are few examples of miRNAs increasing the transcription or translation of target genes [54], [55], resulting in positive correlations between miRNAs and target mRNAs. Therefore, a generally held expectation is that changes in the expression levels of mRNAs will be inversely correlated with changes in the levels of their targeting miRNAs [56]. However, the fact that individual miRNAs may target multiple mRNAs and that individual mRNAs may be targeted by multiple miRNAs creates the potential for a complex network of interactions in cancer cells replete with a variety of positive and negative feedback loops [57], [58]. In addition, the fact that miRNAs are well known to target mRNAs encoding a variety of cellular transcription factors and other regulatory proteins increases the likelihood that the direct regulatory effects of miRNAs may be modulated by indirect or “down-stream” effects within the context of cancer cells. This is not to say that the mechanisms demonstrated to underlie miRNA regulation *in vitro* are inoperative *in vivo*, but rather that the functional consequences of these mechanisms may be masked and/or modulated by the regulatory complexities that characterize cancer cells. The extent to which these complexities may mitigate our ability to accurately predict the molecular consequence of changes in levels of miRNAs within the context of cancer cells has relevance to the potential use of miRNAs in cancer therapy.

The purpose of our study was to evaluate the extent to which the molecular consequences of changes in miRNA levels in cancer cells isolated from patient tissues can be predicted from current models of miRNA function. The fact that prior efforts to obtain expression profiles of miRNAs and their mRNA targets were typically carried out on samples obtained from different patients and/or from mixed (bulk) tissues have contributed to inconsistent findings (e.g., [15], [16], [17], [18], [19], [20]). In an effort to reduce experimental variation, we assayed changes in levels of miRNAs and their targeted mRNAs in ovarian cancer cells isolated from the same patients by LCM. Our results indicate that only ∼11% of the changes in levels of mRNAs are IC with changes in levels of their regulating miRNAs in the same ovarian cancer (CEPI) cells. To eliminate the possibility that our results are merely an artifact of incorrectly identified mRNA targets of miRNA regulation, we repeated our analyses using targets predicted by a variety of prediction algorithms, as well as, targets experimentally validated in a series of transfection experiments conducted in a well-documented ovarian cancer cell line (HEY). Our results consistently support the conclusion that the expected IC between changes in miRNA levels and levels of their target mRNAs occurs relatively infrequently in ovarian cancer cells isolated from patient samples and that this low inverse correlation is not merely an artifact of inaccurate miRNA target predictions. Rather, our results indicate that the low inverse correlation between changes in miRNA levels and levels of their target mRNAs is the likely consequence of indirect cellular processes that modulate or mask the regulatory effects of miRNAs *in vivo*. Our findings underscore the complexities of miRNA-mediated regulation *in vivo* and the need for better understanding the basis of these complexities in cancer cells before the therapeutic potential of miRNAs can be fully realized.

## Materials and Methods

### Tissue samples

Ovarian tumor samples were collected at Northside Hospital (Atlanta, GA) during surgery and snap frozen in liquid nitrogen within 1 minute of removal from patients. All ovarian tumor samples used in this study were from patients diagnosed with serous papillary epithelial ovarian carcinoma. Brushings of normal ovarian surface epithelial cells (OSE) were preserved immediately in RNAlater (Ambion, Austin, TX). Patient consent and approval from the Institutional Review Boards of Georgia Institute of Technology and Northside Hospital were obtained. The written consent and our protocol (#H09227) were approved by the IRB. Detailed clinical information for each patient used in this study is provided in Table 1.

### Laser capture micro-dissection (LCM)

Fresh frozen tissues from tumors were cut into seven-micron sections, applied to non-charged slides, then fixed in 75% ethanol for 30 seconds, stained and dehydrated using the HistoGene LCM Frozen Section Staining Kit (Arcturus, Mountain View, CA). LCM was performed with an AutoPix Automated Laser Capture Microdissection System using the CapSure Macro Caps (Arcturus). Approximately 10,000 cells were captured on each of 5–6 caps per sample. miRNA was extracted from captured cells using the mirVana miRNA Isolation Kit (Ambion). mRNA was isolated from cells from the same patients using the PicoPure RNA Isolation Kit (Arcturus).

### RNA extraction from ovarian surface epithelial cells

For miRNA qPCR and microarray, normal ovarian surface epithelial cells were spun down and resuspended in lysis buffer from the mirVana miRNA Isolation Kit (for small RNA enrichment), following the manufacturer's recommended protocol (Ambion). cDNA for qPCR was synthesized from RNA (10ng) using the TaqMan miRNA Reverse Transcription Kit (Applied Biosystems, Foster City, CA).

For mRNA qPCR and microarray total RNA extraction from OSE was carried out using the PicoPure RNA Isolation Kit, following the manufacturer's recommended protocol (Arcturus). Due to limited number of cells collected from surface epithelial brushings, OSE RNA was extracted from 5 patients with non-malignant ovaries for mRNA microarray and from two *different* sets of three patients with non-malignant ovaries for miRNA (one set for miRNA microarray, another for qPCR) (See Table 1 for more details on patient information).

### Quantitative (real-time) PCR

Total RNA (10ng) extracted from LCM captured ovarian tumor cells and normal OSE cells was converted to amplified cDNA for qPCR. TaqMan miRNA Assays (Applied Biosystems) were conducted following manufacturer's protocol for hsa-miR-141, hsa-miR-429, hsa-miR-205, hsa-miR-320, hsa-miR-383 and for RNU6B endogenous control using the StepOnePlus Real-Time PCR machine (Applied Biosystems). The primer specificities for these miRNAs have been previously demonstrated [58]–[63] and RNU6B is a previously established control for human ovarian tissue (http://www.ambion.com/techlib/tn/151/3.html; http://www3.appliedbiosystems.com/cms/groups/mcb_marketing/documents/generaldocuments/cms_044972.pdf). Statistical significance was determined using the Relative Expression Software Tool (REST; [59]).

For mRNA qPCR, total RNA (1–5 µg) extracted from cells was converted to cDNA using the Superscript III First Strand synthesis system (Invitrogen). cDNA was then purified using the QIAGEN PCR purification kit (QIAGEN) following manufacturer's instructions. qPCR experiments were carried out for the EGFR, BMI1 and GAPDH genes using iQ SYBR Green Supermix (Bio-Rad, Hercules, CA). The sequence specific primers used for SYBR green assays are as follows: EGFR-forward: GGAGAACTGCCAGAAACTGACC, EGFR-reverse: GCCTGCAGCACACTGGTTG, GAPDH-forward: GGTCTCCTCTGACTTCAACA, GAPDH-reverse: AGCCAAATTCGTTGTCATAC, BMI1-forward: ACTTCATTGATGCCACAACC, BMI1-reverse: CAGAAGGATGAGCTGCATAA. The EGFR primers were as designed by [60] and GAPDH primers were as designed by [61]. BMI1 primers were obtained from the qPCR primer database RTPrimerDB [62]. The GAPDH primer efficiencies were calculated to be ∼1. The specificity of the other primers can be obtained from the relevant publications/database. All qPCR reactions (for mRNA and miRNA) were optimized with non-template controls and -RT (minus reverse transcriptase) controls prior to experiment. GAPDH was chosen as endogenous control as it displayed minimal change between cells transfected with miR-NC and miR-7/128 in microarray.

All qPCR reactions were carried out with at least 2 biological replicates and for each biological replicate at least 3 technical replicates.

### Cell culture and Cell Transfections

HEY cells were provided by Gordon B. Mills, Department of Systems Biology, the University of Texas, M. D. Anderson Cancer Center. The cells were cultured in RPMI 1640 (Mediatech, Manassas, VA) supplemented with 10% v/v heat-inactivated fetal calf serum (Invitrogen, Carlsbad, CA), 2 mM L-glutamine (Mediatech), 10 mM HEPES buffer (Mediatech), penicillin (100 U/ml), and streptomycin (100 µg/mL). Approximately 12h before transfection, these cells (duplicates or triplicates per transfection, 1.5×10^5^ per well) were seeded on six-well plates in growth medium and allowed to adhere overnight at 37°C in a 5% CO_2_ atmosphere. The following day after washing the wells with PBS and replacing the growth medium with Opti-MEM (Invitrogen), cells were transfected with the miRNA [hsa-miR-7 miRIDIAN mimic, miRIDIAN miRNA mimic negative control #1 (miR-NC, a *C. elegans* miRNA, cel-miR-67, with confirmed minimal sequence identity in humans), or hsa-miR-128 miRIDIAN mimic (Thermo Fisher Scientific, Lafayette, CO)] using Lipofectamine 2000 transfection agent (Invitrogen) according to the manufacturer's instructions at a final concentration of 25 nM. Cells were incubated for four hours in this reduced serum environment to optimize transfection, washed with PBS, and then incubated at 37°C and 5% CO_2_ for 44 h (total 48 h) after adding fresh growth medium to the wells. Transfection efficiency was estimated from the relative knock-down of previously validated targets (EGFR for miR-7 and BMI-1 for miR-128 [42], [43]), based on recommendations by the siRNA/miRNA reagent manufacturer (Thermo Fisher Scientific). Cell culture experiments were carried out using at least two-three independent biological replicates.

### Microarray

#### Tissue mRNA microarray

Biotin labeled cRNA was synthesized, hybridized to Affymetrix HG-U133 Plus 2.0 oligonucleotide arrays and analyzed with a GeneChip Scanner 3000 (Affymetrix, Santa Clara, CA).

#### HEY cell RNA isolation and mRNA microarray

Total RNA was isolated using the RNeasy Mini RNA isolation kit (QIAGEN, Valencia, CA) according to the manufacturer's instructions. The integrity of the RNA was verified using an Agilent 2100 Bioanalyzer (1.8–2.0; Agilent Technologies, Palo Alto, CA). mRNAs were converted to double stranded (ds)-cDNA and amplified using Applause 3′-Amp System (NuGen, San Carlos, CA). This cDNA was then biotin labeled and fragmented by using Encode Biotin Module (NuGen). The labeled cDNA was hybridized to Affymetrix HG-U133 Plus 2.0 oligonucleotide arrays and analyzed with a GeneChip Scanner 3000 (Affymetrix).

### Microarray data analysis

#### Tissue miRNA microarray data analysis

Samples for our miRNA profiling study were processed by Asuragen Services (Austin, TX), according to the company's standard operating procedures. A custom-manufactured Affymetrix GeneChip® from Ambion was designed to miRNA probes derived from the Sanger miRBase and published reports [24], [25], [26], [63], [64], [65]. Background signal was estimated from antigenomic probe sequences provided by Affymetrix and derived from a larger set of controls used on the Affymetrix human exon array. Spike-in external reference controls were based on non-miRNA control probes that lack homology to the human genome. Arrays within a specific analysis experiment were normalized according to the variance stabilization method described in [66]. A Wilcoxon rank-sum test was used to determine detection calls of the miRNA probe signal compared to the distribution of signals from GC-content matched anti-genomic probes.

A two-sample t-test, with assumption of equal variance, was applied for statistical hypothesis testing. This test defined which probes are considered to be significantly differentially expressed based on a p-value of 0.01 and log_2_ difference ≥1. The signal intensities from these differentially expressed probes were z-score normalized prior to hierarchical clustering.

Unsupervised hierarchical clustering of the samples was performed using Spotfire Decisionsite for Microarray Analysis (DSMA) based on Z-score transformed signal values of all probesets except those with standard deviation <0.5. A “complete linkage” clustering method was employed with “correlation” ‘similarity measure’. Empty values were replaced with the constant 0.

#### Tissue mRNA microarray data analysis

CEL files generated by the Affymetrix Gene Chip Operating System (GCOS) were converted to expression level values using the MAS 5.0 package implemented using the Affymetrix Expression Console software. The log_2_ transformed expression values were then normalized across samples by Z-score calculations using DSMA. Probe sets with “Absent” call in all groups were removed from further statistical analysis. Probe set intensities were filtered with DSMA using a modulation threshold of 1.0 to include only those probe sets with at least a log_2_ expression value of ≥1.0 or fold change ≥2. Differentially expressed probe sets were identified using the t-test function of the Profile ANOVA Tool of DSMA (p<0.005). Annotations for probe sets were obtained from the NetAffx website (http://www.affymetrix.com/analysis/index.affx).

Unsupervised hierarchical clustering of the samples was performed using DSMA based on Z-score transformed signal values of all probesets except those with standard deviation <0.5 and “Absent” call across all samples. A “complete linkage” clustering method was employed with “correlation” ‘similarity measure’. Empty values were replaced with the constant 0.

#### HEY cell mRNA microarray data analysis

Raw data in the form of CEL files were produced by Affymetrix GeneChip Operating System (GCOS) software. Raw data from mRNA microarray were analyzed using the Expression Console software (Affymetrix) and R (www.r-project.org). Normalization was performed using MAS 5.0, PLIER (Expression Console) and GCRMA (R) algorithms. The log_2_ transformed expression values from MAS5.0 were then analyzed for Affymetrix “Present/Absent” calls using DSMA. Probe sets with “Absent” call in all groups were removed from statistical analysis. Average probe set intensities for each group was calculated based on the log_2_ transformed values from PLIER and then filtered with DSMA using a modulation threshold of 0.5 to include only those probe sets with at least a fold change ≥1.4 [log_2_ difference ≥0.5]. The false discovery rate (FDR) for each probe set was calculated from the log_2_ transformed values after GCRMA normalization using the SAM algorithm [67]. Finally, differentially expressed probe sets were identified using a threshold 5% FDR correction, a fold change ≥1.4 and at least “Present/Marginal” call in one sample. These 3 different filtering approaches were used based on recommendations from recent publication by Mieczkowski et al. [68] and the combination of all three was used to achieve the most stringent filtering.

All microarray data from this study are MIAME compliant and have been submitted to GEO under the accession no. GSE23392.

### miRNA target download

The miRNA targets prediction file based on miRanda was downloaded from www.microrna.org (August 2010 release) [34], [35], [36]. Information about the prediction algorithm, parameter settings and raw data source is available on the above link. Only predicted targets with “good” mirSVR score were used. “Good” mirSVR score refers to miRNA targets with <−0.1 score, and “non-good” mirSVR score refers to targets with >−0.1 score obtained from the support vector regression algorithm mirSVR, available with target predictions in the above link. The miRNA targets prediction file based on TargetScan (release 5.1) was downloaded from www.targetscan.org [69], [70], [71]. Targets based on PicTar [72], [73], [74], [75] prediction were also bulk downloaded from the UCSC database. miRNAs for which PicTar predictions were not present in the bulk data file, were manually curated from the tables available on the PicTar web interface (www.pictar.org).

### Predicted target sites analysis

Target sites for 12 differentially expressed miRNAs were selected from the downloaded target prediction files or from the web interface (PicTar). Of those, target sites with Affymetrix “Absent” call in every sample were excluded from further analysis. Each target mRNA was classified as IC, NC or PC depending on the direction of its change in level of expression between OSE and CEPI and that of each targeting miRNA.

Predicted targets of the miRNAs miR-7 and miR-128 identified by miRanda, TargetScan, PicTar and all combinations of the 3 programs were used to calculated IC, NC, and PC fraction of targets in HEY cells transfected by each miRNA (miR-7 or miR-128) based on significance at a fold change threshold of 1.4 and maximum FDR of 5%. Predicted targets that were down-regulated in the transfection experiments were assumed to be “experimentally validated” and the expression patterns of these experimentally validated targets were subsequently used in comparisons between the CEPI and OSE samples (as described above) to identify IC, PC and NC fractions based on significance at a fold change threshold of 2 and t-test p-value <0.005.

## Supporting Information

Figure S1 **Overlap between differentially expressed genes in ovarian cancer and miRNA target genes.** Genomica was used to calculate significant (false discovery rate corrected q<0.05) gene set overlaps between genes differentially expressed in the ovarian cancer data set and gene sets containing targets of individual miRNAs. This is analogous to a “GO” (gene ontology) enrichment analysis (the miRNA identities are the “ontologies” in this case). The coloring represents the percentage (%) of genes within the cancer data set that overlap with the individual miRNA target gene set listed on the right. The miRNAs listed here include differentially expressed miRNAs found in our microarray experiment as well as additional miRNAs predicted using Genomica.
(TIF)

Figure S2 **Unsupervised hierarchical clustering of CEPI and OSE samples based on probesets expressed on the HG-U133 Plus 2.0 array.** An unsupervised hierarchical clustering of the 5 CEPI and 3 OSE samples was carried out using all detected probesets on the HG-U133 Plus 2.0 array, regardless of differential expression. Probesets with standard deviation <0.5 and “Absent” calls across all samples were removed prior to clustering. The clustering shows that the CEPI and OSE samples cluster into separate groups, which suggests the variance between the groups is greater than that within the groups.
(TIF)

Figure S3 **Differentially expressed mRNAs between CEPI and OSE.** Hierarchical clustering of differentially expressed genes between CEPI samples and OSE samples. The ∼3650 probesets correspond to ∼2700 unique gene symbols and were selected based on p-value <0.005, fold change ≥2, and Affymetrix “Present/Marginal” call in at least one sample. The dendogram on the left clusters the up-regulated genes and down-regulated genes into two groups and the number of genes in each of these classes are approximately equal. Gene symbols corresponding to representative differentially expressed probesets are shown on the right (See Table S2 for listing of all differentially expressed probesets).
(TIF)

Figure S4 **Confirmation of successful miR-7 and miR-128 transfection into HEY cells.** Successful transfection of miR-7 and miR-128 in HEY cells (positive control) was confirmed by measuring levels of two previously demonstrated targets of these miRNAs, EGFR and BMI1, by qPCR following transfection of either miR-NC or miR7/miR-128 into HEY cells. The results demonstrate that both BMI1 and EGFR were down-regulated by ∼60% relative to miR-NC (*** p<0.005) after transfection with miR-128 and miR-7 respectively.
(TIF)

Table S1 **Differentially expressed miRNA probesets detected by microarray.** Forty-two differentially expressed miRNA probesets in 3 CEPI and 3 OSE samples as analyzed by microarray (Ambion miRChip V1). These probesets were selected based on a p-value <0.01, log_2_ difference ≥1, and Affymetrix “Present/Marginal” call in at least 1 sample. The mature miRNA names and the sequences corresponding to each probeset, the average log expression values from the OSE and CEPI samples, as well as, the log_2_ difference and t-test p-value calculated from these are given. Probesets that do not refer to miRNAs currently annotated in Sanger miRBase are listed as “exploratory”. Sequences of these exploratory miRNAs are based on computational predictions from previous studies.
(XLS)

Table S2 **Differentially expressed mRNA probes in CEPI compared to OSE.** Differentially expressed mRNA probesets between 3 CEPI samples and OSE from 5 normal samples as analyzed by microarray (Affymetrix HG-U133 Plus 2.0). These probesets were selected based on a p-value <0.005, fold change ≥2, and Affymetrix “Present/Marginal” call in at least one sample. The gene symbols corresponding to each probeset ID, the average log expression values from the OSE and CEPI samples as well as the log_2_ difference and t-test p-value calculated from these are given.
(XLS)

Table S3 **IC, PC and NC targets of miRNAs in tissue samples.** Target predictions from miRanda (M), TargetScan (TS) and PicTar (PT) programs were downloaded (see methods for details) for each of the 12 annotated miRNAs. miRNA targets that were differentially expressed between CEPI and OSE based on t-test p<0.005 and fold change of at least 2 were classified as being IC or PC with their regulating miRNAs (while target genes that do not meet the above criteria are classified as NC). The IC targets are shown in green, PC targets are shown in red, and blue represents NC targets. Targets with “Absent” calls in all samples have been removed. For miR-93*, TargetScan custom (TScustom) (http://www.targetscan.org/vert_50/seedmatch.html) was used to generate TargetScan predictions. For hsa-miR-429 and hsa-miR-93* there were no PicTar predictions and thus targets of these miRNAs were excluded for analysis when calculating intersections.
(XLS)

Table S4 **Summary values of IC, PC and NC targets using TargetScan.** miRNA targets that were differentially expressed between CEPI and OSE based on t-test p<0.005 and fold change of at least 2 were classified as being IC or PC with their regulating miRNAs (while target genes that do not meet the above criteria are classified as NC). Total number of targets from TargetScan algorithm present after removing probesets with “Absent” calls in all samples is shown along with fraction of IC, PC and NC targets for each of the 12 annotated miRNAs. On average, 79.3% of the target mRNAs are NC, 10.4% are inversely correlated and 10.3% are positively correlated with miRNAs.
(XLS)

Table S5 **Summary values of IC, PC and NC targets using PicTar.** miRNA targets that were differentially expressed between CEPI and OSE based on t-test p<0.005 and fold change of at least 2 were classified as being IC or PC with their regulating miRNAs (while target genes that do not meet the above criteria are classified as NC). Total number of targets from PicTar algorithm present after removing probesets with “Absent” calls in all samples is shown along with fraction of IC, PC and NC targets for each of the 12 annotated miRNAs. On average, 80.5% of the target mRNAs are in the NC group, 9.4% are in the IC group and 10.1% are in the PC group.
(XLS)

Table S6 **Summary values of IC, PC and NC targets using overlap of miRanda and TargetScan predictions.** miRNA targets that were differentially expressed between CEPI and OSE based on t-test p<0.005 and fold change of at least 2 were classified as being IC or PC with their regulating miRNAs (while target genes that do not meet the above criteria are classified as NC). Total number of targets from the overlap of miRanda (M) and TargetScan (TS) algorithms present after removing probesets with “Absent” calls in all samples is shown along with fraction of IC, PC and NC targets for each of the 12 annotated miRNAs. On average, 79.2% of the target mRNAs are in the NC group, 9% are in the IC group and 11.8% are in the PC group.
(XLS)

Table S7 **Summary values of IC, PC and NC targets using overlap of miRanda and PicTar predictions.** miRNA targets that were differentially expressed between CEPI and OSE based on t-test p<0.005 and fold change of at least 2 were classified as being IC or PC with their regulating miRNAs (while target genes that do not meet the above criteria are classified as NC). Total number of targets from the overlap of miRanda (M) and PicTar (PT) algorithms present after removing probesets with “Absent” calls in all samples is shown along with fraction of IC, PC and NC targets for each of the 12 annotated miRNAs. On average, 78.3% of the target mRNAs are in the NC group, 8.6% are in the IC group and 13.1% are in the PC group.
(XLS)

Table S8 **Summary values of IC, PC and NC targets using overlap of TargetScan and PicTar predictions.** miRNA targets that were differentially expressed between CEPI and OSE based on t-test p<0.005 and fold change of at least 2 were classified as being IC or PC with their regulating miRNAs (while target genes that do not meet the above criteria are classified as NC). Total number of targets from the overlap of TargetScan (TS) and PicTar (PT) algorithms present after removing probesets with “Absent” calls in all samples is shown along with fraction of IC, PC and NC targets for each of the 12 annotated miRNAs. On average, 76.8% of the target mRNAs are in the NC group, 6.4% are in the IC group and 16.7% are in the PC group.
(XLS)

Table S9 **Differentially expressed genes between miR-7 transfected and negative control transfected HEY cells.** Differentially expressed genes (fold change ≥1.4, 5% FDR) in miR-7 transfected cells compared to miR-NC transfected cells. ‘Probeset ID’ refers to Affymetrix HG-U133 Plus 2.0 probeset identifier. ‘Gene Symbol’ shows the official gene symbol for the corresponding Probeset ID. ‘log difference (miR7-miR-NC)’ refers to the difference between average log_2_ signal values of miR-7 transfected group and the miR-NC transfected group. ‘q-value (%)’ shows the false discovery rate calculated using the SAM algorithm. Targets of miR-7 predicted by miRanda (M), TargetScan (TS), and PicTar (PT) programs are also shown.
(XLS)

Table S10 **Differentially expressed genes between miR-128 transfected and negative control transfected HEY cells.** Differentially expressed genes (fold change ≥1.4, 5% FDR) in miR-128 transfected cells compared to miR-NC transfected cells. ‘Probeset ID’ refers to Affymetrix HG-U133 Plus 2.0 probeset identifier. ‘Gene Symbol’ shows the official gene symbol for the corresponding Probeset ID. ‘log difference (miR128-miR-NC)’ refers to the difference between average log_2_ signal values of miR-128 transfected group and the miR-NC transfected group. ‘q-value (%)’ shows the false discovery rate calculated using the SAM algorithm. Predicted targets from miRanda (M), TargetScan (TS), and PicTar (PT) are also shown.
(XLS)
