# Supplementary figures and images for: Evidence for the Complexity of MicroRNA-Mediated Regulation in Ovarian Cancer: A Systems Approach
Source: PLoS One. 2011 Jul 21;6(7):e22508. doi: 10.1371/journal.pone.0022508 (PMC3141058; doi:10.1371/journal.pone.0022508)

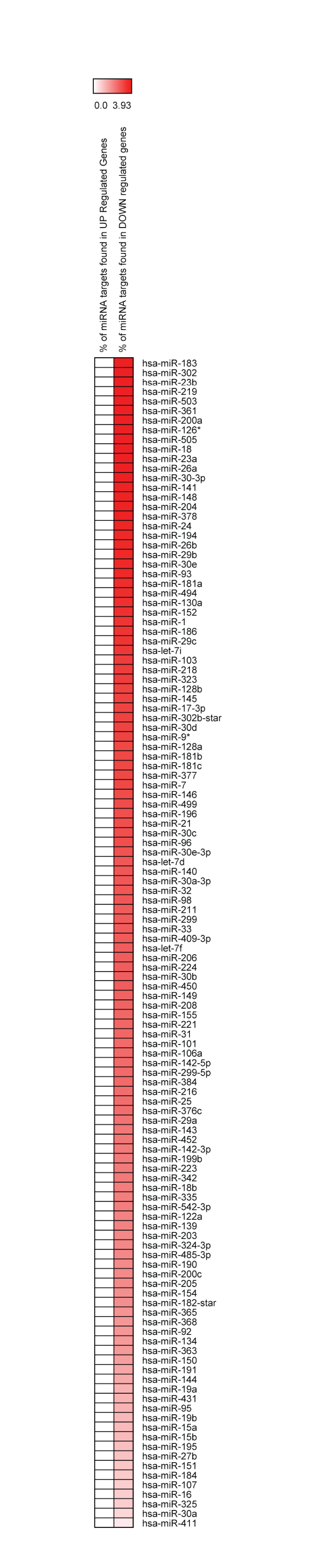

Supplement: Figure S1 — Overlap between differentially expressed genes in ovarian cancer and miRNA target genes. Genomica was used to calculate significant (false discovery rate corrected q<0.05) gene set overlaps between genes differentially expressed in the ovarian cancer data set and gene sets containing targets of individual miRNAs. This is analogous to a “GO” (gene ontology) enrichment analysis (the miRNA identities are the “ontologies” in this case). The coloring represents the percentage (%) of genes within the cancer data set that overlap with the individual miRNA target gene set listed on the right. The miRNAs listed here include differentially expressed miRNAs found in our microarray experiment as well as additional miRNAs predicted using Genomica. (TIF) [file pone.0022508.s001.tif]

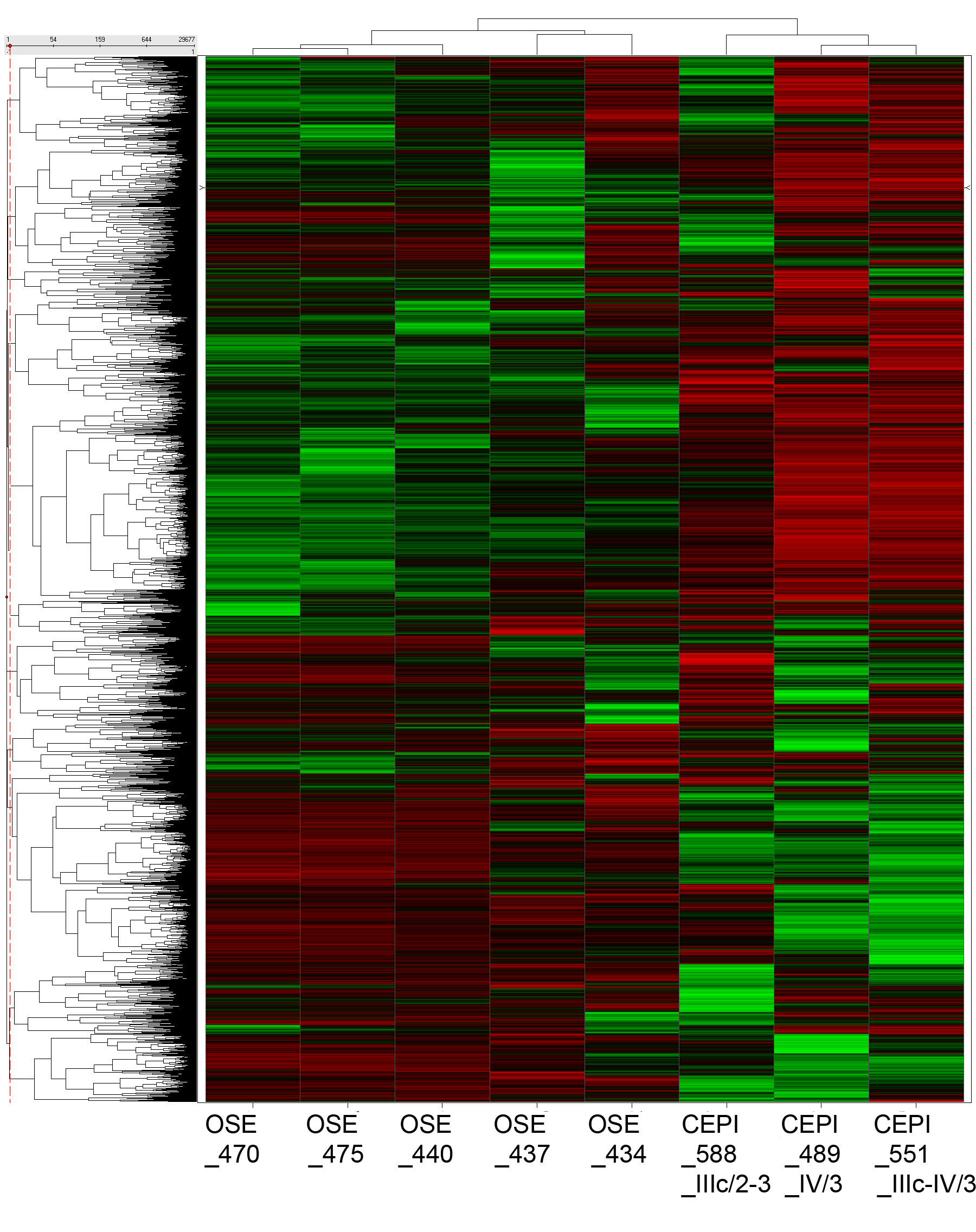

Supplement: Figure S2 — Unsupervised hierarchical clustering of CEPI and OSE samples based on probesets expressed on the HG-U133 Plus 2.0 array. An unsupervised hierarchical clustering of the 5 CEPI and 3 OSE samples was carried out using all detected probesets on the HG-U133 Plus 2.0 array, regardless of differential expression. Probesets with standard deviation <0.5 and “Absent” calls across all samples were removed prior to clustering. The clustering shows that the CEPI and OSE samples cluster into separate groups, which suggests the variance between the groups is greater than that within the groups. (TIF) [file pone.0022508.s002.tif]

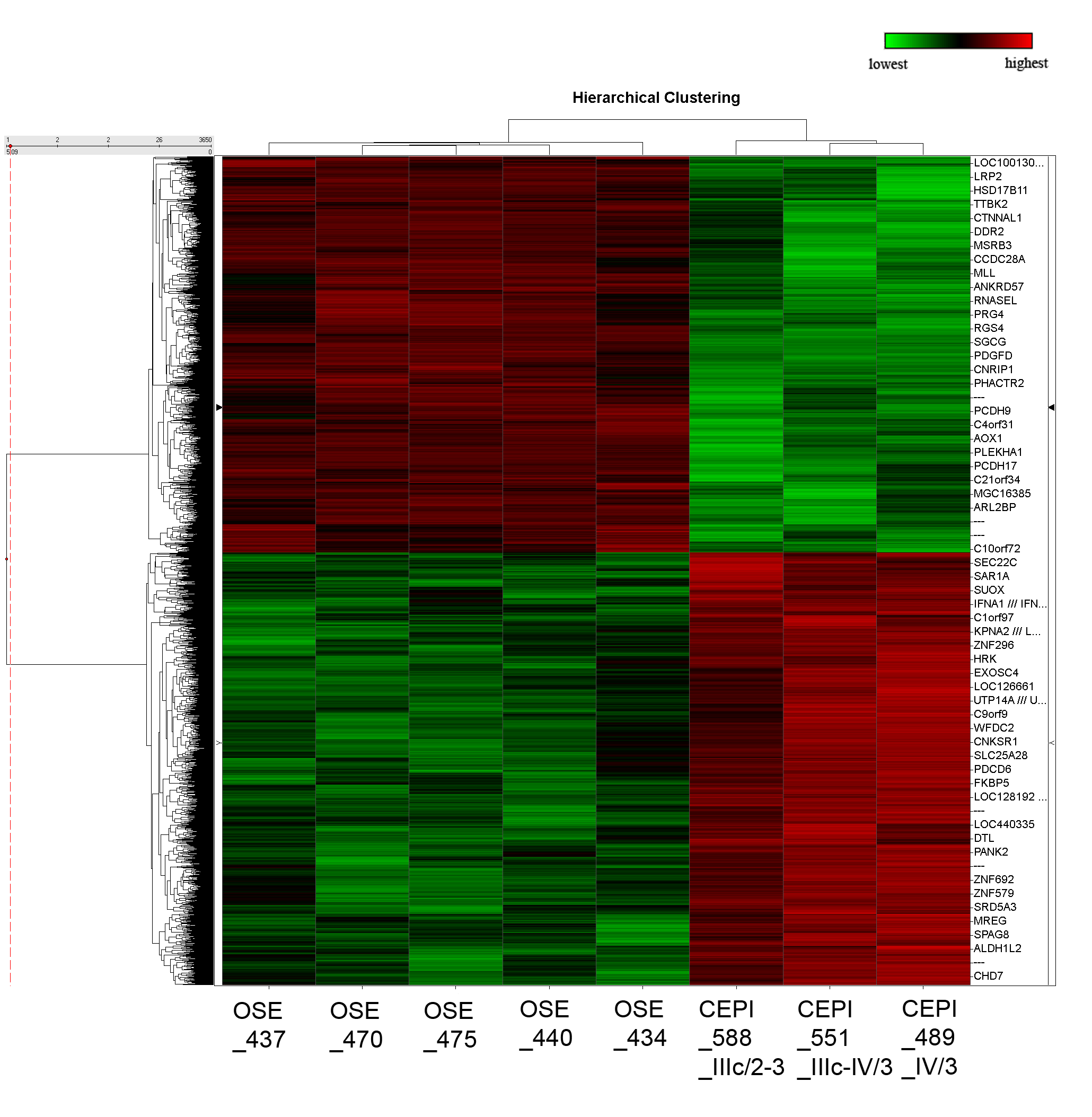

Supplement: Figure S3 — Differentially expressed mRNAs between CEPI and OSE. Hierarchical clustering of differentially expressed genes between CEPI samples and OSE samples. The ∼3650 probesets correspond to ∼2700 unique gene symbols and were selected based on p-value <0.005, fold change ≥2, and Affymetrix “Present/Marginal” call in at least one sample. The dendogram on the left clusters the up-regulated genes and down-regulated genes into two groups and the number of genes in each of these classes are approximately equal. Gene symbols corresponding to representative differentially expressed probesets are shown on the right (See Table S2 for listing of all differentially expressed probesets). (TIF) [file pone.0022508.s003.tif]

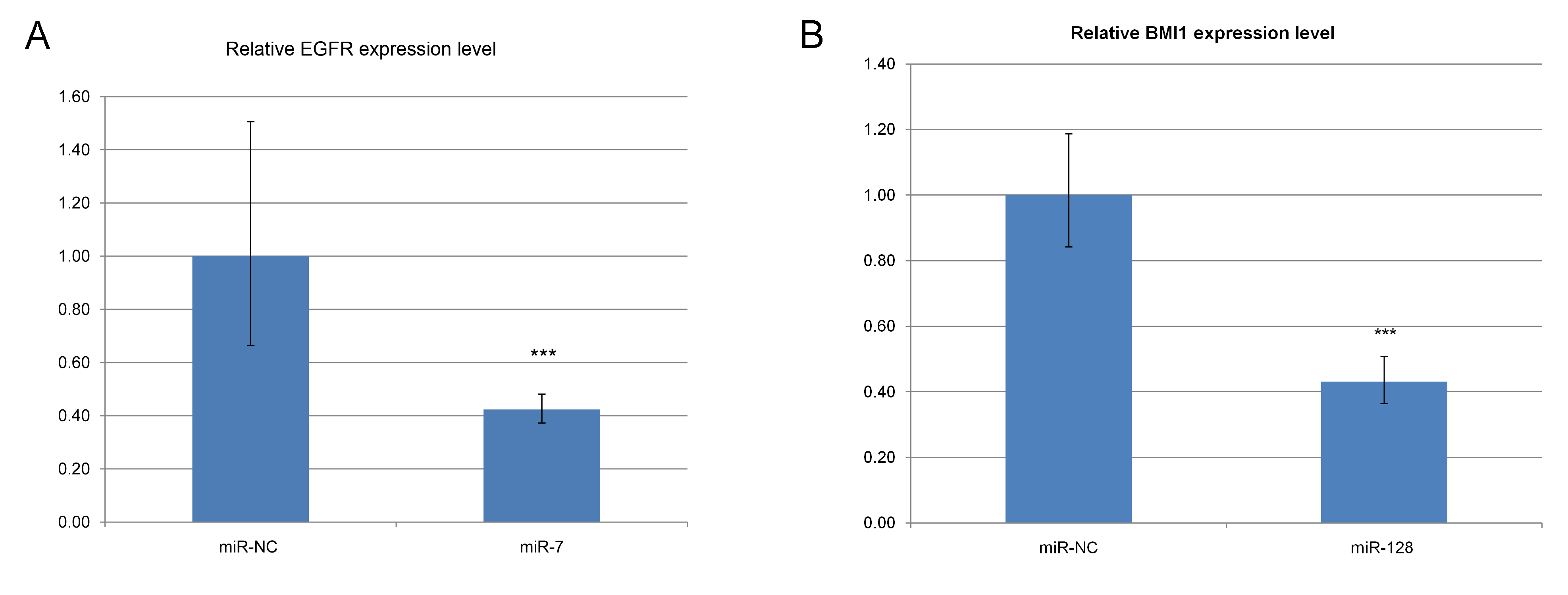

Supplement: Figure S4 — Confirmation of successful miR-7 and miR-128 transfection into HEY cells. Successful transfection of miR-7 and miR-128 in HEY cells (positive control) was confirmed by measuring levels of two previously demonstrated targets of these miRNAs, EGFR and BMI1, by qPCR following transfection of either miR-NC or miR7/miR-128 into HEY cells. The results demonstrate that both BMI1 and EGFR were down-regulated by ∼60% relative to miR-NC (*** p<0.005) after transfection with miR-128 and miR-7 respectively. (TIF) [file pone.0022508.s004.tif]
